# Perceptions and underpinnings of climate justice demands: investigating the public’s conception of international and individual-level duties

**DOI:** 10.3389/fpsyg.2026.1897314

**Published:** 2026-09-08

**Authors:** Kirsti M. Jylhä, Göran Duus-Otterström, Krister Bykvist

**Affiliations:** 1 Institute for Futures Studies, Stockholm, Sweden; 2 Department of Political Science, University of Gothenburg, Gothenburg, Sweden; 3 Department of Philosophy, Stockholm University, Stockholm, Sweden

**Keywords:** climate justice, climate policy, environmental attitudes, justice perceptions, policy support, public perceptions, climate action

## Abstract

Issues of climate justice are extensively debated among scholars and politicians, yet research into public perceptions is scarce. In a Swedish study (*N* = 680), we examined public perceptions, intercorrelations, and correlates of various debated climate justice issues, here referred to as “climate justice demands.” Our main analyses focused on two indexes: (1) international-level demands: *on behalf of poor countries*, based on different climate justice principles (polluter pays, ability to pay, right to development), and; (2) individual-level demands: *individual duties to act*, including views on private/generalized morality, and interpersonal fairness. We found widespread support for demands for rich and high-emitting countries to provide climate aid to poor countries, and for individual climate action. In regression analyses, after controlling for all covariates, both our indexes correlated negatively with climate change denial, and positively with climate worry on behalf of more distant others (future generations, people living in poorer countries, and animals/nature), personal climate action, and political trust. Moreover, international-level demands correlated positively with age, and negatively with personal climate worry and social dominance orientation, while individual-level demands correlated negatively with (male) gender. The results of our study could be considered in climate change communication. It seems possible that at least some opposition to climate policies could be reduced by explicitly connecting them to justice principles. Considering the persistent gridlock in international and domestic climate politics, we call for more research to examine climate justice perceptions across societal groups and cultural contexts. Our interdisciplinary analyses, combining psychology, political science, and philosophy, could guide such inquiries.

## 1 Introduction

Most people across the world want more effective climate action and policy (Bouman et al., 2024; Flynn et al., 2024), yet many of the suggested mitigation and adaptation measures are both debated and opposed (Ekberg et al., 2022). At the core of these debates are issues of justice, encompassing a wide range of considerations related to fairness and morality in the causes, consequences, and solutions of climate change (Parsons et al., 2024; Tschötschel et al., 2025).

Climate justice can mean very different things to different people, resulting in diverging views about how the climate crisis should be tackled (Piispa and Kiilakoski, 2022). The variety of climate justice considerations is reflected in the persistent gridlock in climate change policy, as the public, interest groups, and nations disagree on who should act, why, and how (see also Carlsson et al., 2013; Schleich et al., 2016). Thus, understanding views about climate justice can help explain why certain climate actions and policies may be supported or opposed, and aid effective and fair policy making (Clayton et al., 2013; Montada and Kals, 2000; Pearson et al., 2021). Despite this, relatively few studies have examined public perceptions about climate justice. Some justice considerations have received increasing attention, such as the harmful consequences that are disproportionately felt among the most vulnerable and least responsible groups (e.g., Carman et al., 2025), the fairness of domestic climate policies such as carbon taxes (e.g., Maestre-Andrés et al., 2019), and individual duties to act (e.g., Leviston and Walker, 2021). However, several other justice perceptions – including questions about distribution of costs between countries – have been largely ignored, despite their potential to shape attitudes toward environmental action. There is also a lack of studies that examine intercorrelations between, and underpinnings of, different climate justice perceptions. This hinders our understanding of the sources of disagreement among the public. There is a particularly urgent need for interdisciplinary work that bridges empirical work with the rich theoretical accounts on climate justice.

In this paper, using nationally representative data from Sweden, we examine public perceptions on responsibilities and obligations at international and individual levels, which we refer to as *climate justice demands*. Building on our respective disciplines, our analyses combine theoretical frameworks, analytic tools, and empirical approaches from psychology, political science, and philosophy. Our main focus is on two categories of perceptions: (1) *demands to provide climate aid for poor countries*, based on different climate justice principles (polluter pays, ability to pay, right to development), and; (2) *individual duties to act on climate change*, including views on private/generalized morality and interpersonal fairness. These issues are commonly debated in climate negotiations, and among political philosophers and environmental activists (Caney, 2018; Martiskainen et al., 2020; Moellendorf, 2012; Tschötschel et al., 2025). Yet not much is known about how the public thinks about them. Thus, this paper examines the prevalence, intercorrelations, and potential correlates of different international and individual-level justice demands in the Swedish public. For comparative purposes, and to provide a more comprehensive understanding of climate justice perceptions, we also examine views on climate refugee migration, emission permissions for poor countries, and the question of who should act (individuals and/or governments/industries).

A related concept to climate justice is that of the just transition, which may be understood as justice in the transformation, or pathway, to a low-carbon society. Debates over this concept overlap with debates around climate justice in several respects, but they sometimes focus on distinctive issues having to do with, e.g., the restructuring of the labor market and ways of dealing with job loss (Newell and Mulvaney, 2013; Wang and Lo, 2021). Here, we do not seek to contribute to the latter discussion but rather investigate the public’s perception of climate responsibilities in a broader sense. However, since a just transition must involve a just distribution of costs and responsibility between relevant actors, our results remain relevant for most debates taking place under the rubric of “just transition.” The observation that some political opposition or gridlock can be explained by diverging standards of justice apply here too since the transition to a low-carbon society involves difficult tradeoffs where different conceptions of justice will come to different conclusions (Newell and Mulvaney, 2013).

### 1.1 Climate justice

Justice, in its most encompassing sense, concerns what people are due. Different kinds of justice can be specified by identifying the relevant group of people, what they are due and on what grounds, and who is responsible for making sure people get what they are due. An especially important distinction is between *distributive* and *corrective* justice. Principles of distributive justice seek to distribute benefits and burdens according to some just criterion, such as equality, efficiency, or need. In contrast, principles of corrective justice are about rectifying past wrongdoings through remedies such as compensation, disgorgement, or punishment.

Discussions about climate justice largely proceed against the backdrop of the distributive/corrective distinction.^1^ Internationally, the major question concerns “burden sharing”: how the costs, sacrifices, or efforts of responding to climate change ought to the shared between countries. To limit the scope of this paper, our empirical focus is particularly on the question of burden sharing in assisting poor countries in adaptation and mitigation. We further ask whether poor countries can be exempted from burden sharing if this would help them develop economically. These questions are relevant due to multiple reasons. Poor countries have contributed least to climate change, yet they are particularly vulnerable to its effects (Althor et al., 2016; Roberts and Parks, 2007; Schlosberg and Collins, 2014). Further, they lack the necessary resources to securely adapt to the changing climate, which is a serious problem particularly in counties located in the Global South, as they face the most serious weather hazards.

When theorizing burden sharing, some endorse corrective principles such as *the polluter pays principle*, stressing that costs should be borne by those that primarily caused the problem (see e.g., Tan, 2023). Others, however, reject the image of climate change as one involving wrongdoing for which corrective remedies are due (see e.g., Posner and Weisbach, 2010). This is because, for a long time, climate change was caused under conditions of excusable ignorance and without the right kind of regulatory environment (Schüssler, 2011; Bou-Habib, 2019). Some argue that we can accommodate these points while preserving a fundamentally corrective understanding of climate justice by adopting *the beneficiary pays principle*. On a prominent version of this view, past emissions should be seen as a source of unjust enrichment and those who are currently sitting on those benefits owe it to others to pay for climate policy (Page, 2012). Those who endorse taking an essentially forward-looking approach to climatic burden sharing – one that foregrounds distributive justice rather than focusing on previous wrongdoing – frequently endorse *the ability to pay principle*, which states that costs should be assigned to those with the broadest financial shoulders (Roser and Seidel, 2017). Still others argue that the correct response is to search for mixed, or hybrid, positions which balance corrective and distributive concerns, especially a country’s record of emitting and its current ability to pay (Vanderheiden, 2008; Caney, 2010; Page, 2011).

Related to the debate about burden sharing is the debate about international emissions rights (Schulan et al., 2023). The historically most prominent view is to defend an equal per capita share of the overall emissions (carbon) budget combined with emissions trading (Torpman, 2019). Others, however, stress that emissions permits must be allocated based on need, giving strong priority to securing opportunities to generate *subsistence emissions*, which are emissions necessary for meeting basic human needs (Shue, 1993; Duus-Otterström, 2023). This is closely connected to the idea that emissions rights should be seen through the lens of a right to development. Many have argued that countries may have a special right to emit based on the need to develop out of poverty (Shue, 1993; Moellendorf, 2014). There is an emerging debate whether the right to generate subsistence emissions, or to develop out of poverty, conflicts with not only an egalitarian distribution of emissions permits but also the idea of a predefined emissions budget (Duus-Otterström, 2024; Tank, 2024). A related debate concerns whether the history of the problem matters in the sense that countries that have already profited from fossil fuel emissions owe it to other countries to enable the same emissions opportunities or remove the need for them via transfer of green energy technology (Agarwal and Narain, 1991; Shue, 2019).

The debate about international climate justice is largely mirrored in the debate over *individual obligations*. The focus here has mainly been on whether individuals have a moral duty to reduce their own emissions (Fragnière, 2016). Some defend a restrictive account, where individuals should seek to avoid all but the most essential emissions since emissions contribute to harm (Hiller, 2011; Tank, 2024). Others argue that individuals should instead ensure that emissions are kept within one’s fair share (Vanderheiden, 2016; Torpman, 2019). Still others argue that individuals lack obligations to emit less altogether due to the inconsequential nature of their individual contribution to the climate problem (Sinnott-Armstrong, 2010). Instead, on this view, the individual’s obligation is primarily to cooperate with others to pursue effective political solutions (Cripps, 2013; Maltais, 2013). This view is consistent with an obligation to mitigate insofar as an individual’s mitigation makes a difference for collective action (Cripps, 2013; Hohl and Roser, 2011).

### 1.2 Climate justice perceptions among the public

Environmental justice is a central theme for environmental activism and movements (e.g., Bührle and Kimmerle, 2021; Martiskainen et al., 2020; Piispa and Kiilakoski, 2022). Despite this, relatively few studies have studied perceptions about climate justice in the public. In this limited research, the focus has mostly been on the disproportional impacts of climate change on vulnerable countries and human communities (e.g., Carman et al., 2025; Whitmarsh and Corner, 2017). Some studies have additionally considered responsibilities to non-human animals and the rights of the environment (Clayton, 2000; Reese and Jacob, 2015), procedural justice (Carman et al., 2025; Ogunbode et al., 2024), or certain political aspects, such as the role of the capitalist system in driving climate change (Ogunbode et al., 2024). The findings have generally shown that the public widely recognizes and expresses concern for such justice issues.

Related to justice considerations, previous research has also examined perceptions of fairness in certain domestic environmental policies, particularly carbon taxes (e.g., Bergquist et al., 2022; Levain et al., 2022). The results highlight that one of the most common reasons to oppose such climate policies is their perceived unfairness (Maestre-Andrés et al., 2019; Povitkina et al., 2021).

Importantly, however, only a few studies have examined perceptions of justice in international climate policy (see Meilland et al., 2024; Trott et al., 2023). To our knowledge, the most comprehensive study was conducted by The Finnish Climate Change Panel, revealing strong support for several climate justice considerations (Vainio et al., 2023). For example, it showed that a clear majority of the respondents thought that high-emitting countries should bear the costs of mitigation, and that industrialized countries should offer adaptation aid for developing countries (Vainio et al., 2023). In line with this, the Peoples’ Climate Vote 2024-survey included a question to measure support for rich countries giving more help to poorer countries to address climate change (Flynn et al., 2024). It found widespread agreement across 77 countries. One line of research has aimed to examine how people rank climate justice principles in the context of domestic emission reductions or allocations of mitigation costs (Carlsson et al., 2011, 2013; Meilland et al., 2024; Schleich et al., 2016), or whether highlighting some justice-related factors (past emissions, economic resources, or solidarity) might influence environmental attitudes, including the attitude toward helping people displaced by climate change (Anderson et al., 2017; Charnysh et al., 2024; Klebl and Stanley, 2025). While these studies are valuable, more research taking a wider scope on international climate justice perceptions is needed.

When it comes to views about individual engagement, the topic has not been studied using the terminology of climate justice *per se*, but some justice-related views have been examined. These include, firstly, the question of individual responsibility to act. The results have shown that people tend to place more responsibility on governments and organizations than individuals, although it is not uncommon to also report a sense of personal responsibility or to demand action from other people (e.g., Andrews et al., 2025; Eurobarometer, 2021; Leviston and Walker, 2021). Secondly, there is a fair bit of research about perceived morality of climate engagement. The results have generally shown that many people see climate change as a moral issue capable of generating moral obligations (e.g., Doran et al., 2019; Leviston and Walker, 2012; Lorenzoni and Pidgeon, 2006). Previous research has typically not addressed the more nuanced views about morality, for example the role of reciprocity in influencing demands to act on climate change (Jagers et al., 2020). Thus, in this paper, we examine views about individual’s climate duties in a more systematic way than usually done in literature.

### 1.3 What explains climate justice perceptions?

Climate justice perceptions vary across societal groups, creating conflicting views about environmental protection and policy legitimacy (Meilland et al., 2024). There are various potential sources of this variation.

First, sociodemographic factors, such as social class, gender, and age, can entail conflicting interests and risk perceptions. For example, socio-economic status can influence views about economic costs of mitigation or adaptation, and age has important implications for personal risk scenarios and vulnerability to climate change (see, e.g., Gregersen et al., 2025; Poortinga et al., 2019). Further, women tend to respond to environmental issues with greater concern than men, for example due to gender differences in vulnerability to environmental hazards, and personality dispositions and socialization practices that entail caring, nurturing, and empathetic orientation toward other people and nature (see, e.g., Blocker and Eckberg, 1989; Davidson and Freudenburg, 1996; Milfont and Sibley, 2016).

Second, ideological views influence perceptions of climate justice. Right leaning and conservative ideologies tend to be more accepting of prevailing socioeconomic structures and social inequalities (e.g., Duckitt, 2001). This could generate opposition against structural and lifestyle changes to protect the environment (e.g., Clarke et al., 2019; Feygina et al., 2010) as well as engender more tolerance for social injustice in climate change (e.g., Jackson et al., 2013; Jylhä, 2016; Jylhä et al., 2021). Indeed, left-leaning and socially liberal views are associated with stronger concerns of social justice and morality in climate change (Clayton, 2013; Currie and Choma, 2018; Ogunbode et al., 2024; Schuldt and Pearson, 2023), and responsiveness to climate justice messages (Charnysh et al., 2024; Whitmarsh and Corner, 2017), while right-leaning and conservative views are associated with perceiving national climate policies as unjust and unfair (Gregersen et al., 2025; Vainio et al., 2023).

Third, climate policy support has been linked to political trust (Bergquist et al., 2022; Fairbrother et al., 2021). Perceptions of policy makers and political institutions as incompetent and corrupt can make people question the legitimacy of policies, particularly if these policies entail personal risks, extended costs, or sacrifice (Citrin and Stoker, 2018; Devine, 2024).

Fourth, pro-environmental concerns and attitudes correlate with awareness and concern for the disproportional negative impacts that the less advantaged and less responsible populations face due to changing climate (e.g., Ogunbode et al., 2024), perceived moral duty and obligation to act on climate change (Doran et al., 2019; Leviston and Walker, 2012; Steg et al., 2011), and perception that various actors (groups, organizations, individuals) have the responsibility to address climate change (Leviston and Walker, 2021).

While the literature has explained some of the variation in climate justice perception, to our knowledge, no research has examined the intercorrelations and correlates of justice demands in international burden sharing or compared the correlates of international- and individual level climate justice demands. This is what we aim to do in this paper.

### 1.4 Aims and hypotheses

This paper examines public perceptions on climate justice demands in Sweden. Our first two aims are to study the prevalence (*aim 1*) and intercorrelations (*aim 2*) of these climate justice demands. To this end, we formulated survey items to measure perceptions of responsibilities and obligations in international policy and individual action. The international-level items were intended to capture demands to assist poor countries. Our battery of questions is designed to allow us to triangulate the relative support for principles based on a country’s record of emitting or ability to pay, and views on poor countries’ right to develop, supported either by climate aid or emissions permissions. Individual-level demands, meanwhile, were intended to capture views about individual duties to act. These items included views on private and generalized morality of climate action and interpersonal fairness.

Based on previous research reviewed above, we expected to find support for the demand that rich and high-emitting countries should provide aid for poor countries and that individuals should act on climate change (Hypothesis 1). However, demand to allow poor countries prioritize economic growth could be more difficult to assess, and thus our analyses with this item were exploratory.

Our third aim is to study the correlations of both our main categories of justice demands (international and individual) with a set of potential predictor variables: climate-related concerns and behaviors (climate change denial, worry, and action), sociopolitical attitudes (conservative attitudes, political trust) and demographic factors (age, education level, gender). Building on above-reviewed previous research that has studied correlates of attitudes toward social justice (including in the climate context) and environmental issues, we expected that support for the items capturing both international and individual-level climate justice demands correlate with the predictor variables included (Hypothesis 2). Our analyses also include regression analyses to estimate the distinct and combined effects of our predictor variables in explaining variance in climate justice demands. Due to scarcity of previous research on the topic, these analyses are exploratory and we did not formulate hypotheses.

Finally, in addition to the above-described categories of items, we included also questions about climate refugee migration, unfairness regarding economic sacrifice in respondents’ own country, and individual and governmental/industry responsibilities. We analyze these items for comparative purposes and with the purpose of offering a more complete understanding of climate justice perceptions. However, these analyses are only briefly presented and discussed, to ensure theoretically consistent and parsimonious main analyses.

## 2 Materials and methods

### 2.1 Procedure and participants

Data collection was conducted in November 2022 by the professional research company Lysio Research. The sample was nationally representative in age, gender, and geographic distribution. The questionnaire was completed online and took approximately 20 min to complete. Participants were rewarded with points that correspond to 20 SEK and could be chosen to be paid out as money or donated for charitable purposes. The sample consisted of 680 participants, with ages ranging between 18 and 91 (*M* = 49.7, SD = 19.1). 50.6 percent reported themselves as female, 49.1 percent as male, and 0.3 as other (handled as missing value). An additional three respondents participated but were excluded from the analyses due to unreliable response patterns. The distribution of education level was the following: elementary school or equivalent: 8%; high school or equivalent: 30%; education after high school (excl. university): 12%; studies at university: 18%; university degree: 32%; other 0.4%. The study was conducted adhering to the national ethical legislation and the ethical guidelines specified by the Swedish Research Council and the American Psychological Association.

### 2.2 Measures

#### 2.2.1 Climate justice demands

We formulated 12 survey items to measure climate justice demands, introduced with the passage; “There are many different views on justice and ethics when it comes to climate change. As is often the case with such issues, there are no clear answers as to what is right or wrong. You will now be presented with a number of statements about climate justice, and we ask you to indicate the extent to which you agree with them.” Participants responded to each of these items on a five-point scale ranging from 1 (“Strongly Disagree”) to 5 (“Strongly Agree”), or “don’t know” (handled as missing value). As presented below, the items were examined within the categories international- and individual-level demands.

##### 2.2.1.1 International-level demands

Six items, introduced in Table 1, aimed at triangulating the support for different international climate justice demands. Our main focus was on the first four items. Items 1 and 2 capture support for the polluter pays principle (stressing emissions as the basis for a duty) and the ability to pay principle (stressing wealth). As for item 3, this is worded such that support is either indicative of support for the ability to pay principle or enlightened self-interest (stressing the benefits of helping poor countries grow economically in a sustainable way). This item was included to assess whether it is particularly strongly supported compared to items 1 and 2, potentially suggesting “additional” effect of stressing co-benefits of assistance (mitigation in poor countries). Item 4 was designed to measure a different value conflict than the one about offering assistance, in this case the one stemming from recognition of both our collective duties to contribute to climate change mitigation and a right to development in poor countries.

**TABLE 1:** Item wording, descriptive statistics [mean value (M) and standard deviation (SD), scale range 1–5] of, and correlations between, the items measuring climate justice demands at international level.

Survey item	1	2	3	4	5	*M*	*SD*
1. The countries that emit the most should help poor countries deal with the negative impacts of climate change.*	–	–	–	–	–	4.10	0.97
2. Rich countries should help poor countries deal with the negative impacts of climate change.*	**0.62**					4.08	0.96
3. Rich countries should help poor countries grow economically in ways that do not damage the environment.*	**0.51**	**0.69**				4.13	0.96
4. Poor countries should not have to reduce their emissions if this would hinder their economic development	0.05	**0.08**	**0.09**			2.29	1.06
5. Sweden should not have to reduce emissions if this would hinder our economic development.	**−0.28**	**−0.39**	**−0.40**	**0.25**		2.32	1.13
6. Sweden should offer asylum to people who need to leave their homes due to climate change.	**0.35**	**0.42**	**0.41**	**0.12**	**−0.41**	2.86	1.29

Statistically significant (*p* < 0.05) correlations are **bolded**; For exact *p*-values, see Supplementary Table 4; *Items included in the index “Demands on behalf of poor countries.”

Note that these four items are specifically worded in terms of a duty to “help poor countries.” We follow the convention of treating the effects of climate change in poor countries as especially worthy of attention, the assumption being that rich countries are more able to handle negative effects, and decouple their economic growth from emissions, on their own. Thus, responses to these items indirectly capture the degree to which respondents agree with international distributive obligations.^2^ From the perspective of poor countries, these three questions capture ability to pay principle and right to development principles.

In addition to the above-described items focusing on demands on behalf of poor countries, we also measured support for accepting climate refugee migration (item 5) and perceptions of unfairness regarding the need for one’s country to prioritize climate policies over economic development (item 6). Throughout our survey, we did not say what it means for a country to be “rich” or “poor”; we left it to the respondents to apply their own standard here. We assume, however, that most respondents will have assumed that Sweden is a rich country

##### 2.2.1.2 Individual-level demands

Six items, introduced in Table 2, referred to demands for individual duties of justice. Items 7 and 8 are included to measure whether people feel personally morally responsible, and tend to see climate change as a moral issue for individuals to begin with. Items 9 and 10 were formulated to capture conditional vs. non-conditional sense of responsibility to limit one’s emissions. Item 9 offers a direct measure of support for reciprocity, item 10 measures the extent to which lack of reciprocity is seen as unfair. We also measured perceptions of the climate problem as a responsibility of individuals or entities like governments and industry (items 11–12)

**TABLE 2:** Item wording, descriptive statistics [mean value (M) and standard deviation (SD), scale range 1–5] of, and correlations between, the items measuring climate justice demands at individual level.

Survey item	7	8	9	10	11	*M*	*SD*
7. I feel morally responsible for limiting my personal contribution to climate change.*	–	–	–	–	–	3.70	1.14
8. Individuals’ climate behaviors reveal their moral character.*	**0.44**					3.25	1.08
9. If other people reduce their emissions, I should do the same.*	**0.50**	**0.37**				4.20	0.96
10. It is not unfair if I must change my lifestyle to reduce my emissions if others don’t do the same^†^	**0.33**	**0.11**	**0.15**			3.29	1.28
11. Individuals are not to blame for climate change.	**−0.21**	**−0.21**	**−0.13**	**−0.17**		3.14	1.15
12. Tackling climate change is mainly the responsibility of governments and industries.	**−0.09**	0.06	−0.02	**−0.16**	**0.19**	3.36	1.10

The items are numbered to continue from Table 1, to facilitate clearer discussion about the full set of items; Statistically significant (*p* < 0.05) correlations are **bolded**; For exact *p*-values, see Supplementary Table 5.

*Items included in the index “Demands for individual action.”

† The original item did not include “not”; added here because the item is reversed to ease interpretations.

##### 2.2.1.3 Indexes

As described more closely in the section “3.2 Intercorrelations between justice demands,” we created two indexes that capture international-level demands (assisting poor countries) and individual-level demands (obligations and duties). These are denoted as “Justice demands on behalf of poor countries” (items 1–3: α = 0.82, *M* = 4.09, SD = 0.84) and “Demands for individual action” (items 5–7: α = 0.70, *M* = 3.73, SD = 0.86).

#### 2.2.2 Predictor variables

The survey also included measures to potential correlates of climate justice demands. Participants responded to each of these items by five-point scales. The full list of items and response scales are presented in the Supplementary Material.

To measure climate worry, we modified a scale from Ojala (2012) and divided it into two indexes: personal climate worry (on behalf of participants themselves and their close ones; α = 0.83, *M* = 3.09, SD = 1.02) and distant climate worry (on behalf of future generations, people living in poorer countries, and animals/nature; α = 0.85, *M* = 3.77, SD = 0.99)^3^. Personal climate action was measured by engagement in 12 different forms of private-sphere and social actions (Jylhä et al., 2025: α = 0.84, *M* = 2.87, SD = 0.70). Climate change denial was measured by a six-item scale, capturing denial of the trend, human causes, and negative consequences of climate change (Jylhä et al., 2025: α = 0.88; *M* = 1.87, SD = 0.83). Political trust was measured by asking the degree to which participants trust “the national parliament” and “politicians” (α = 0.85, *M* = 2.66, SD = 0.94). Conservative attitudes were measured using two scales that capture (1) Social Dominance Orientation (SDO: support for social group-based hierarchies: Pratto et al., 1994) with the four-item Short (SSDO)-scale (Pratto et al., 2013: α = 0.71, *M* = 1.90, SD = 0.75), and (2) Right-Wing Authoritarianism (support for strong authorities and adherence to tradition: Altemeyer, 1981) with three questions from the scale developed to the Swedish context (Zakrisson, 2005), earlier used for example by Jylhä et al. (2025: α = 0.43, *M* = 2.85, SD = 0.76).

## 3 Results

### 3.1 Prevalence of agreeing with climate justice demands

We first examined the level of agreement with the different items. Mean values and standard deviations are presented in Tables 1, 2. The prevalence of agreement and disagreement are presented in Supplementary Tables 1, 2 ^4^.

Overall, as illustrated in Table 1, the questions regarding demands to provide aid for poor countries (items 1–3) yielded strong support among participants. A clear majority agreed with these items at least partly (76%–80%). However, as for the demand to allow exempting poor countries from emissions reduction (item 4), only a minority stated that this should be allowed for the sake of economic growth (14%). Moreover, only a minority supported the demands to accept climate refugees (31%) or to prioritize economy over mitigation in Sweden (16%).

As for individual-level justice demands, the items expressing individual duties were generally supported (see Table 2). A majority agreed they have an obligation to reduce their emissions if other people do the same (78%) and reported feeling morally responsible to act on climate change (63%). Moreover, a sizable minority agreed that climate behaviors reveal individuals’ moral character (42%). A minority considered it to be unfair to be asked to change their lifestyle if others don’t do the same (30%), or agreed that individuals are not to blame (36%), and about half agreed that mitigation is mainly the responsibility of government and industries (52%).

### 3.2 Intercorrelations between justice demands

We then addressed our second aim and examined correlations between the items measuring climate justice demands. Here, we examined associations between our items within their relevant categories (international or individual level, see Tables 1, 2).

#### 3.2.1 International-level demands

We found positive correlations between items that capture justice demands related to poor countries (items 1–3 in Table 1), and these items correlated positively with the demand to accept climate refugees. The items measuring demand to permit emissions for the sake of economic development correlated positively with each other. However, when agreement of permitting emissions was asked in the Swedish context, the correlations with other variables were negative, while positive (and relatively weak) correlations were observed when asked considering poorer countries.

Examining the intercorrelations further, we conducted an exploratory factor analysis (Principal axis factoring, Direct Oblimin, missing cases replaced with mean), which resulted in two factors. In combination with the correlation analyses (Table 1), the results suggest that the first three items could be combined in an index (factor loadings 0.66–0.86). We thus created an index of them, denoted as “Demands on behalf of poor countries.” The remaining items were not combined into indexes due to conceptual incompatibility, weak or inconsistent factor loadings, and analyses of reliability scores (Cronbach’s alpha).

#### 3.2.2 Individual-level demands

All four items representing support for individual duties of justice correlated positively with each other (see Table 2), but correlations tended to be weaker with the question capturing conditional duties (item 4, reversed in the correlation analyses). We note that these items can be described as capturing individual and generalized morality, and interpersonal fairness. These items correlated negatively with the view that individuals are not to blame, while inconsistent and mostly relatively weak correlations were found with thinking that governments and industries bear the main responsibility.

We then conducted an exploratory factor analysis which revealed two factors. In line with the correlations reported above, the first factor consisted of the three first items supporting demands for individuals (factor loadings 0.60–0.72). We thus created an index of these questions, denoted as “Demands for individual action”^5^. Even though the remaining items loaded on the second factor, we did not compose an index of these questions because the intercorrelations (Table 2) and factor loadings were relatively weak.

### 3.3 Correlations with attitudinal and demographic variables

We then examined the correlates of each item measuring different aspects of climate justice demands, starting with international-level questions. As shown in Table 3, the items capturing demands to aid poor countries (items 1–3) correlated – with almost no exceptions – positively with personal and distant climate worries, climate action, political trust, age, and education level. Moreover, these items correlated negatively with climate change denial, and conservative attitudes (SDO and RWA). Similar correlations were found with accepting climate refugees (item 6), and also (male) gender correlated with this item (negatively). Opposite correlation patterns were observed when examining agreement with allowing emissions for the sake of economic development in poor countries and Sweden (items 4 and 5). However, many of the correlations with the formerly mentioned item were relatively weak and statistically non-significant.

**TABLE 3:** Correlations between attitudinal and demographic variables and climate justice demands at international level.

	1. The countries that emit the most should help poor countries deal with the negative impacts of climate change.	2. Rich countries should help poor countries deal with the negative impacts of climate change.	3. Rich countries should help poor countries grow economically in ways that do not damage the environment.	4. Poor countries should not have to reduce their emissions if this would hinder their economic development	5. Sweden should not have to reduce emissions if this would hinder our economic development.	6. Sweden should offer asylum to people who need to leave their homes due to climate change.
Climate worry, personal	**0.17**	**0.18**	**0.19**	**−0.11**	**−0.28**	**0.22**
Climate worry, distant others	**0.40**	**0.44**	**0.42**	−0.04	**−0.44**	**0.35**
Climate change denial	**−0.46**	**−0.52**	**−0.53**	**0.10**	**0.55**	**−0.40**
Climate action	**0.29**	**0.40**	**0.35**	−0.02	**−0.44**	**0.50**
Political trust	**0.14**	**0.14**	**0.17**	**0.13**	**−0.10**	**0.15**
Social Dominance Orientation (SDO)	**−0.37**	**−0.46**	**−0.41**	0.04	**0.37**	**−0.42**
Right-Wing Authoritarianism (RWA)	**−0.22**	**−0.26**	**−0.27**	−0.07	**0.36**	**−0.43**
Age	**0.13**	**0.18**	**0.20**	**−0.12**	**−0.13**	**−0.02**
Gender (male)^†^	0.01	−0.02	−0.07	0.01	**0.14**	**−0.14**
Education level^†^	0.07	**0.10**	**0.12**	0.03	**−0.14**	**0.14**

Statistically significant (*p* < 0.05) correlations are **bolded**. For exact *p*-values, see Supplementary Table 6;

† Spearmans Rho, other correlations: Pearson.

As for correlates of individual-level justice demand (see Table 4), three main items capturing supporting views regarding individual duties of justice (items 7–9) correlated positively with personal and distant climate worries, climate action, political trust, age, and education level. Further, these items correlated negatively with climate change denial, conservative attitudes (SDO and RWA), and (male) gender. Most of these correlations were statistically significant. Similar correlations were found with perceiving no unfairness in action demands in the case of other people’s inaction (item 10, the item was reversed). Examining questions of rejecting the blame on individuals (item 11), we observed negative correlations with personal and distant worries, and climate action, and positive correlations with climate change denial, conservative attitudes (SDO), and (male) gender. As to the question of governmental/industry obligations (item 12), only one correlation – the negative correlation with age – was statistically significant.

**TABLE 4:** Correlations between attitudinal and demographic variables and climate justice demands at individual level.

	7. I feel morally responsible for limiting my personal contribution to climate change.	8. Individuals’ climate behaviors reveal their moral character.	9. If other people reduce their emissions, I should do the same.	10. It is *not unfair if I must change my lifestyle to reduce my emissions if others don’t do the same.	11. Individuals are not to blame for climate change.	12. Tackling climate change is mainly the responsibility of governments and industries.
Climate worry, personal	**0.31**	**0.26**	**0.23**	0.04	**−0.21**	0.06
Climate worry, distant others	**0.49**	**0.32**	**0.40**	**0.23**	**−0.20**	0.04
Climate change denial	**−0.52**	**−0.31**	**−0.46**	**−0.27**	**0.20**	**−**0.06
Climate action	**0.54**	**0.39**	**0.41**	**0.31**	**−0.17**	**−**0.05
Political trust	**0.17**	0.03	**0.19**	**0.17**	**−**0.05	0.01
Social Dominance Orientation (SDO)	**−0.37**	**−0.16**	**−0.31**	**−0.24**	**0.09**	**−**0.01
Right-Wing Authoritarianism (RWA)	**−0.29**	**−**0.03	**−0.20**	**−0.28**	0.03	0.01
Age	0.01	**0.09**	**0.08**	**0.14**	**−**0.02	**−0.12**
Gender (male) ^†^	**−0.24**	**−0.11**	**−0.14**	**−0.17**	**0**.**10**	0.03
Education level^†^	**0.17**	0.02	**0.13**	**0.12**	**−**0.02	**−**0.01

Statistically significant (*p* < 0.05) correlations are **bolded**. For exact *p*-values, see Supplementary Table 7;

† Spearmans Rho, other correlations: Pearson.

*The original did not include “not”; added here because the item is reversed to ease interpretations.

In sum, people who are more concerned and engaged with the climate issue, politically trusting, less conservative, older, female, and more educated tend to be more supportive of climate justice demands on behalf of internationally worst off and regarding individual moral obligations. They also tend to be less supportive of allowing emissions to grow in Sweden. Three of the questions were relatively weakly – if at all – correlated with the attitudinal and demographic variables: allowing emissions to grow for the sake of economic development in poor countries, rejecting individual responsibility, and governmental/industry responsibility.

### 3.4 Regression analyses

We then examined the unique and combined contributions of the included predictor variables in explaining variance in our international-level index (justice demands on behalf of poor countries) and individual-level index (justice demands for individuals). In a series of regression analyses, we tested four models where these two indexes were placed as dependent variables. As shown in Table 5, the models included demographic variables (Model 1), sociopolitical attitudes (Model 2), climate-related views and behaviors (Model 3), or all variables (Model 4). No serious concerns were detected regarding multicollinearity assumptions (Tolerance scores: 0.49–0.95).

**TABLE 5:** Regression analyses examining the standardized effects (beta values) of attitudinal and demographic variables on climate justice demands at international and individual levels.

	Demands on behalf of poor countries		Demands for individual action	
	Adjusted R^2^	β	Adjusted R^2^	β
Model 1	0.056		0.063	
Age		0.20***		0.10*
Gender (male)		−0.07		−0.22***
Education level		0.12**		0.11**
Model 2	0.259		0.147	
Political trust		0.14***		0.12**
SDO		−0.44***		−0.31***
RWA		−0.08*		−0.09*
Model 3	0.401		0.428	
Climate worry, personal		−0.13***		0.04
Climate worry, distant		0.25***		0.17***
Climate change denial		−0.43***		−0.28***
Climate action		0.14***		0.33***
Model 4	0.458		0.436	
Age		0.14***		0.05
Gender (male)		0.05		**−**0.09**
Education level		0.03		0.02
Political trust		0.11***		0.08**
SDO		−0.21***		−0.02
RWA		0.03		0.04
Climate worry, personal		−0.09*		0.02
Climate worry, distant		0.20***		0.17***
Climate change denial		−0.35***		−0.28***
Climate action		0.11**		0.31***

SDO, Social Dominance Orientation; RWA, Right-Wing Authoritarianism; For *b*-values (including 95% confidence intervals) and exact *p*-values, see Supplementary Table 8. *p < 0.05, **p < 0.01, ***p < 0.001.

The results showed that, in three models, the included sets of variables explained a comparable share of variance in both indexes: demographic variables explained 6 and 7 percent (Model 1), climate-related views and behaviors explained 40 and 43 percent (Model 3), and the full model explained 46 and 44 percent (Model 4), respectively. A larger difference was found in the model including sociopolitical attitudes (Model 2), which explained more variance in the international-level demands (26%) than in the individual-level demands (15%).

We then examined the unique effects of the predictor variables on our two indexes. In Model 1, age and education had positive and statistically significant effects on both indexes, while (male) gender only had an effect (negative) on individual-level demands. In Model 2, political trust had a positive effect on both indexes, and SDO and RWA had negative effects. In Model 3, both climate worry on behalf of distant others and personal climate action had positive effects on both indexes, and climate change denial had negative effects. However, personal climate worry had a statistically significant effect only on international-level demands, and this effect was negative, differing from the zero-order correlational patterns which indicated a positive correlation (see Table 3). Closer examination of this correlation revealed that the variable that changed the direction of personal worries (from β = 0.20 to β = −0.10) was, when being controlled for, worries on behalf of more distant others. Finally, the results of Model 4 show that, when controlling all the variables, climate change denial was a relatively strong predictor of both indexes. Personal climate action was also relatively strongly correlated with individual-level demands but weakly correlated with international-level demands. A unique share of variance in both indexes was also explained by climate worries about distant others and political trust. In addition to these results, international-level demands were predicted by age, SDO (low), and personal worry (low), while individual-level demands were predicted by gender (female). Thus, when comparing with the zero-order correlation patterns and Models 1–2, some effects became non-significant: the effect of RWA and education on both indexes, and the effect of SDO on individual-level demands.

## 4 Discussion

Issues of climate justice are extensively debated among scholars and politicians, yet little is known about what the public thinks. This paper focused on the public’s perception of different principles of burden sharing between countries – with a particular focus on demands on behalf of poor countries, as well as the manner and extent to which respondents extend obligations of climate justice to individual people. In general, we found widespread support for aiding the most vulnerable, although less support was found for accepting climate refugees. These demands correlated with pro-environmental attitudes, concerns, and behavior, and some variables that have been identified as key predictors of environmentalism in previous research (e.g., political trust and less conservative attitudes). Some results contradicted our hypotheses, most importantly the positive correlation between age and support for climate justice demands. Next, we discuss the results in more detail.

### 4.1 Support for climate justice demands

Our first aim was to study the prevalence of agreement on various justice demands. Consistent with previous research focusing on social justice in the context of climate change (see, e.g., Schleich et al., 2016; Ogunbode et al., 2024), respondents widely supported the demands for climate engagement and allocations of burdens to wealthier and most emitting countries.

Items 1–3 (regarding aid to poor countries) garnered virtually identical support. This suggests two important conclusions. First, it suggests that the respondents indeed see these policies as justice demands rather than prudential strategy, considering that item 3 (worded to highlight co-benefits of mitigation in poor countries) did not generate significantly higher support than items 1–2 (worded to highlight adaptation aid). This is why we describe the index comprising items 1–3 as “justice demands on behalf of poor countries.” Second, the respondents appear to make no meaningful distinction between the polluter pays principle (item 1) and the ability to pay principle (item 2). While we cannot exclude the possibility that these principles are seen as equally plausible, the results could suggest that the public do not recognize them as independent principles, at least not in their extension (i.e., they think rich countries are high emitters and vice versa). More research is needed to examine if these conclusions are correct, for example by further disentangling the relative weight of corrective justice and distributive justice in the public’s climate policy opinions.

Regarding the value conflict related to economic development and emissions reductions, it is noteworthy that the results do not support any general preference for environmentally detrimental development. Instead, we found a strong commitment to emission reductions even in poor countries. This goes against much of the philosophical discussion in academic literature, which tends to stress a right to emit to develop out of poverty (see e.g., Shue, 1993; Moellendorf, 2014). However, our results should not be interpreted as suggesting low support for poverty eradication. Instead, we suspect it should rather be seen as indicative of support for indirect mitigation. This interpretation is plausible considering the widespread agreement with the idea that rich countries should help poor countries to grow economically in environmentally sustainable ways (item 3). This is also in line with previous research showing that people tend to place high priority on both poverty eradication and climate change mitigation (e.g., Eurobarometer, 2021).

Regarding individual-level demands, we observed strong general support for individual action, although a slight majority indicated that the government and industries bear the main responsibility to mitigate climate change. This is in line with previous research results showing that, while most responsibility tends to be placed on politicians and industries, it is also common to demand that individuals, including the respondents themselves, act on climate change (e.g., Eurobarometer, 2021). Thus, people do not seem to consider the responsibilities of individuals and governments/industries to be competing in the way that they are sometimes depicted in public discussions (see also Climate Outreach, 2021).

### 4.2 Intercorrelations between climate justice demands

Our second aim was to study intercorrelations between perceptions of different climate justice demands. We found that some of the questions correlated with each other strongly enough to be combined into indexes. This conclusion was further supported by their shared correlational patterns with the included set of covariates. We thus created two indexes: First, ‘demands on behalf of poor countries’ includes items supporting climate aid for poor countries, based on three climate principles (polluter pays, ability to pay, and right to development). Second, “demands for individual action” includes questions about individual morality, generalized morality, and interpersonal fairness.

The need for standardized surveys on climate justice has been stressed recently (Meilland et al., 2024). We argue that our scales and individual items could be used in future research. Yet, considering the various justice considerations across different responses to climate change, we acknowledge that our study – in combination with other research (e.g., Gregersen et al., 2025; Ogunbode et al., 2024) – can be only seen as a starting point. More systematic intercultural and interdisciplinary research is needed before valid and reliable scales can be constructed. We left unaddressed, for example, procedural justice, as well as some prominent principles of distributive justice, such as the beneficiary pays principle (Roser and Seidel, 2017). Moreover, perceptions on justice principles can vary depending on their applications across various different domestic or international policies. Collaborations across disciplines are needed to combine the rich theoretical considerations (e.g., political theory, philosophy, psychological processes) and empirical work.

One starting point for scale development could be to ask what the public can be expected to form an opinion about. A recent review of climate justice communications found that the literature has most addressed the justice issues related to the impacts of climate change, followed by questions about responsibilities, often in terms of international distribution (Tschötschel et al., 2025). Nevertheless, some scales could be developed to examine intuitions regarding questions that are less familiar to the public. One line of research could also test whether detailed descriptions of the justice principles influence how they are perceived.

It is also important to consider our survey items in relation to general practices of measuring environmental attitudes. As reviewed by Hadler et al. (2021), scales vary vastly in terms of the measured aspects, for example in their focus on hypothetical or actual behaviors, principled or concrete policy proposals, or low-impact or high-impact climate action. Most our items were based on considerations about rather abstract climate justice principles, and future research could aim to develop scales that also capture views about what these principles entail in terms of concrete policies and their expected impacts on mitigation/adaptation, and social justice. It would also be beneficial to consider possible structural and political conditions for climate action in society (see Hadler et al., 2021), as this could influence how justice principles are perceived.

### 4.3 Correlates of climate justice demands

The third aim was to investigate the correlates of climate justice perceptions. For more parsimonious discussion, we focus here on the regression analyses on the two indexes. Justice demands at both international and individual level were more strongly supported by people who are more convinced about climate change (express low denial), personally engaged in action, concerned on behalf of distant others (future generations, poor people, animals/nature), and politically trusting. The other patterns differed between the two indexes, with individual-level demands being connected with (female) gender, and international-level demands being connected with Social Dominance Orientation (low), and age. It is also noteworthy that self-reported climate action was more strongly connected with individual than international-level demands. This supports the notion that people aim to act in accordance with their personal values, see their actions as moral, and morally disengage with actions that conflict their morality (in the climate context, see e.g., Nicolai et al., 2022; Steg, 2023).

In the full model, some effects vanished: The effect of Right-Wing Authoritarianism on both indexes, and the effect of Social Dominance Orientation on the individual-level index. This happened when controlling for climate-related variables (e.g., climate change denial), which could perhaps be expected considering that conservative attitudes and anti-environmentalism are correlated in the Western context (see, e.g., Hornsey et al., 2016; Stanley et al., 2024). Interestingly, Social Dominance Orientation was the stronger predictor of both indexes in the model that included sociopolitical attitudes (Model 2). This variable also had a unique and strong effect on international-level demands in the full model. Thus, acceptance of group-based social hierarchies seems to be relevant variable explaining variance in climate justice demands, particularly in the context of demanding aid to poor countries.

Considering the correlations with demographic variables, gender was not linked to international-level demands but had a unique effect on individual-level demands even when other variables were controlled for. Gender is consistently linked to various pro-environmental attitudes and behaviors (e.g., Poortinga et al., 2019) and is also central to issues of climate justice (e.g., McKinney and Fulkerson, 2015). Women are disproportionally more harmed by environmental hazards compared to men and are more concerned about environmental issues. Thus, gender equality and female participation in political sphere can significantly improve the prospects of just and effective climate change mitigation (McKinney and Fulkerson, 2015). However, gender is more consistently connected to concerns over local environmental hazards than to general environmental concern (e.g., Blocker and Eckberg, 1989), and this could perhaps explain why we did not find correlations between gender and international-level demands. These results are consistent with studies that have not found gender to be connected to climate justice perceptions and beliefs (Gregersen et al., 2025; Ogunbode et al., 2024). Yet another noteworthy finding was that age correlated positively with climate justice demands. This was unexpected, considering that it is usually suggested – and in some research empirically shown – that younger people are particularly aware and concerned about climate justice (Gregersen et al., 2025; Ogunbode et al., 2024; Schuldt and Pearson, 2023). Our results perhaps differ because our study did not focus on the same aspects of climate change that previous research has studied, such as the disproportional harm that climate change is causing to vulnerable populations.

We also observed that the correlation with personal climate worries changed direction from positive to negative when controlling for distant worries. Future research could examine whether personal worries are in part motivated by self-interest, and whether distant worries are more strongly linked to justice-related perceptions and/or empathetic concern.

### 4.4 Limitations and cultural context

Our study was one of the first to examine climate justice perceptions among the public. Combining our respective fields, we draw from political theory, climate ethics, and environmental psychology, to design a study that captures various relevant climate justice considerations and their potential psychological underpinnings.

However, some limitations in our study should be mentioned. One limitation is that we cannot make conclusions about causality. Experimental and longitudinal research designs could test how attitudes regarding climate justice demands develop, and whether such attitudes influence social and environmental views and behaviors.

Another limitation is that our study did not address the ambivalence many people may feel regarding climate justice demands. Environmental action in general tends to be characterized by ambivalent attitudes and emotions, such as perceiving climate action as both important and ineffective (Van Gent et al., 2024). There is no universal agreement regarding what justice principles should be followed in general, and the context of climate change adds additional layer of moral disagreement due to the collective responsibilities and competing goals that individuals and nations may have (see e.g., Asker, 2023).

The cultural context is also relevant. Most research on climate justice has been conducted in Western context (e.g., Europe, USA, Australia, but see e.g., Andrews et al., 2025; Ogunbode et al., 2024), and our study is no exception, limiting possibilities to draw any universal conclusions. Moreover, Sweden is a relatively liberal, egalitarian, wealthy, and environmentally conscious country, and it could thus be expected that people widely think that Sweden has suitable structural conditions for climate action, and the responsibility and economic resources to help others (see also Lindvall et al., 2025). Thus, it is not surprising that we observed widespread perception that climate action is a moral issue and obligation. Future research could further examine the prevalence and correlates of climate justice perceptions both within and between nations of varying levels of economic development and vulnerability to climate change.

## 5 Conclusion

We found strong support for individual climate action and for climate aid to poor countries from rich and high-emitting countries. This support could be considered when formulating and justifying climate policies. It seems possible that at least some opposition to policies such as carbon taxes and strict emission restrictions in affluent countries could be reduced by explicitly connecting them to justice principles. We call for more research to systematically examine perceptions of the various issues of climate justice across societal groups and cultural contexts. Considering the persistent gridlock in international climate negotiations and societal action, there is a need for increased understanding of climate justice perceptions among the public. Our study, bridging empirical work with rich theoretical accounts on climate justice and psychology, could help moving forward further academic inquiries into this topic.

## Data Availability

The raw data supporting the conclusions of this article will be made available by the authors, without undue reservation.

## Footnotes

1 Another important dimension, which we do not discuss here, is procedural justice. For a valuable overview of academic debates over climate justice, see Caney (2021).

2 If we would had stricken the phrase “in poor countries,” item 3 would have offered a way to measure support for a purely corrective understanding of climate justice where a high-emitting country may owe assistance to a rich country as compensation. However, given the standard north-south framing of the climate issue, we suspect that respondents would still have assumed that the recipients of help would be poor and vulnerable countries.

3 These two indexes, measured with similar questions, are called micro and macro-worries by Wullenkord and Ojala (2023). To highlight worries relevant in the climate justice context, our distinction differentiates personal worries from worries on behalf of more distant others. This is of importance in the context of climate justice, as these more distant others also belong to groups that are least responsible for and most vulnerable to climate change.

4 We also examined the prevalence of the two categories of climate worries (see Supplementary Table 3). Only 8 percent were very worried about being personally affected by climate change, and 14 percent were worried on behalf of their friends and/or family. Thus, it was clearly more common to be very worried on behalf of more distant others: future generations (36%), animals and/or nature (37%), or people who live in poorer countries (25%).

5 We also tested the correlation between the two indexes, and found them to be strongly intercorrelated, *r* = 0.51, *p* < 0.001.
