# Supplementary material for: Perceptions and underpinnings of climate justice demands: investigating the public’s conception of international and individual-level duties
Source: Front Psychol. 2026 Sep 8;17:1897314. doi: 10.3389/fpsyg.2026.1897314 (PMC13593410; doi:10.3389/fpsyg.2026.1897314)
Supplement: Supplementary file 1 [file Supplementary_file_1.docx]

Supplementary Material

**Perceptions and underpinnings of climate justice demands: Investigating the public’s conception of international and individual-level duties**

# 1 Questionnaires

*NB: Originally in Swedish. The questions were randomized within each scale. Reversed items marked with (R).*

***Climate worry, personal and on behalf of more distant others***

Please indicate the extent to which you are concerned about the following scenarios regarding the possible negative impacts of climate change.

- That I myself will be affected
- That my friends and/or my family will be affected
- That future generations will be affected
- That animals and/or nature are affected
- That people living in poorer countries are affected

Scale: 1=Not at worried; […] 5=Very worried

***Personal climate actions***

Indicate the degree to which you have engaged in the following behaviors that have been described as measures to reduce climate impact.

- Avoid flying
- Avoid driving/travelling by a car
- Avoid eating meat
- Avoid dairy products
- Recycle (e.g., metal, plastic)
- Avoid food waste
- Buy green energy
- Reduce use of electricity
- Purchase carbon offsets
- Vote a party/politician that focus on the climate issue
- Participate in demonstrations (e.g., Fridays for future)
- Discuss climate change with friends and family

Scale: 1=Not at all; […] 5=To high degree; 6= Not applicable

***Climate change denial***

- It is true that global warming is happening. (R)
- It is unclear if the earth's climate is changing.
- Global warming that is caused by humans is happening. (R)
- The variation in the Earth's temperature is natural, and is not due to human activities .
- Climate change will not have a notable impact on humans’ living conditions in any significant way.
- Climate change will cause widespread losses and damages for people. (R)

Scale: 1=Strongly Disagree; […] 5=Strongly Agree; 6=Don’t know

***Political trust***

To what degree do you personally trust the following institutions and societal actors?

- The national parliament
- Politicians

Scale: 1=Not at all; […] 5=To high degree

***Social Dominance Orientation***

- In setting priorities, we must consider all societal groups. (R)
- We should not push for equality of societal groups.
- The equality of societal groups should be our goal. (R)
- Superior societal groups should dominate inferior groups.

Scale: 1=Strongly Disagree; […] 5=Strongly Agree; 6=Don’t know

***Right-Wing Authoritarianism***

- To stop the radical and immoral currents in society today there is a need for a strong leader.
- Our society would be best off if we showed tolerance and understanding for non-traditional values and views. (R)
- The best way to live is in accordance with the old-fashioned values.

Scale: 1=Strongly Disagree; […] 5=Strongly Agree; 6=Don’t know

# 2 Supplementary Tables

| **Table S1.**  *Prevalence (%) of disagreeing or agreeing with the international-level climate justice demands.* | | | | | |
| --- | --- | --- | --- | --- | --- |
|  | Strongly  Disagree | Partly  Disagree | Neither Agree nor Disagree | Partly  Agree | Strongly  Agree |
| 1. The countries that emit the most should help poor countries deal with the negative impacts of climate change. | 2.5 | 3.3 | 17.9 | 34.2 | 42.2 |
| 2. Rich countries should help poor countries deal with the negative impacts of climate change. | 2.7 | 4.0 | 13.8 | 41.4 | 38.1 |
| 3. Rich countries should help poor countries grow economically in ways that do not damage the environment. | 2.2 | 4.2 | 14.2 | 37.4 | 41.9 |
| 4. Poor countries should not have to reduce their emissions if this would hinder their economic development | 26.7 | 33.9 | 25.5 | 11.5 | 2.5 |
| 5. Sweden should not have to reduce emissions if this would hinder our economic development. | 29.1 | 29.7 | 25.3 | 12.0 | 3.9 |
| 6. Sweden should offer asylum to people who need to leave their homes due to climate change | 19.7 | 19.0 | 29.9 | 18.1 | 13.3 |

| **Table S2.**  *Prevalence (%) of disagreeing or agreeing with the international-level climate justice demands.* | | | | | |
| --- | --- | --- | --- | --- | --- |
|  | Strongly  Disagree | Partly  Disagree | Neither Agree nor Disagree | Partly  Agree | Strongly  Agree |
| 7. I feel morally responsible for limiting my personal contribution to climate change. | 6.6 | 7.4 | 22.8 | 36.0 | 27.3 |
| 6. Individuals’ climate behaviors reveal their moral character. | 9.7 | 9.0 | 39.4 | 30.7 | 11.1 |
| 9. If other people reduce their emissions, I should do the same. | 2.4 | 2.1 | 17.5 | 29.4 | 48.6 |
| 10. It is unfair if I must change my lifestyle to reduce my emissions if others don't do the same. | 21.4 | 26.2 | 23.0 | 19.1 | 10.4 |
| 11. Individuals are not to blame for climate change. | 7.7 | 21.5 | 34.5 | 21.4 | 14.9 |
| 12. Tackling climate change is mainly the responsibility of governments and industries. | 5.9 | 17.6 | 24.5 | 38.6 | 13.4 |

| **Table S3.**  *Prevalence (%) of worry, answering the question “Please indicate the extent to which you are concerned about the following scenarios regarding the possible negative impacts of climate change.”* | | | | | | |
| --- | --- | --- | --- | --- | --- | --- |
|  | Not at all worried | 2 | 3 | 4 | Very worried |  |
| That I myself will be affected | 13.2 | 20.6 | 38.2 | 20.1 | 7.8 |  |
| That my friends and/or my family will be affected | 6.8 | 14.9 | 34.4 | 29.7 | 14.3 |  |
| That future generations will be affected | 4.1 | 9.7 | 18.1 | 31.8 | 36.3 |  |
| That animals and/or nature are affected | 3.5 | 10.6 | 19.6 | 29.3 | 37.1 |  |
| That people living in poorer countries are affected | 5.1 | 11.2 | 28.4 | 30.7 | 24.6 |  |

| **Table S4**.  *Correlations/p-values between the items measuring climate justice demands at international level.* | | | | | |
| --- | --- | --- | --- | --- | --- |
|  | 1 | 2 | 3 | 4 | 5 |
| 1. The countries that emit the most should help poor countries deal with the negative impacts of climate change. | - |  |  |  |  |
| 2. Rich countries should help poor countries deal with the negative impacts of climate change. | .62/<.001 | - |  |  |  |
| 3. Rich countries should help poor countries grow economically in ways that do not damage the environment. | .51/<.001 | .69/<.001 | - |  |  |
| 4. Sweden should offer asylum to people who need to leave their homes due to climate change | .35/<.001 | .42/<.001 | .41/<.001 | - |  |
| 5. Sweden should not have to reduce emissions if this would hinder our economic development. | -.28/<.001 | -.39/<.001 | -.40/<.001 | -.41/<.001 | - |
| 6. Poor countries should not have to reduce their emissions if this would hinder their economic development | .05/.177 | .08/.040 | .09/.026 | .12/.004 | .25/<.001 |

| **Table S5**.  *Correlations/p-values between the items measuring climate justice demands at individual level.* | | | | | |
| --- | --- | --- | --- | --- | --- |
|  | 7 | 8 | 9 | 10 | 11 |
| 7. If other people reduce their emissions, I should do the same. | - |  |  |  |  |
| 8. I feel morally responsible for limiting my personal contribution to climate change. | .50/<.001 | - |  |  |  |
| 9. Individuals’ climate behaviors reveal their moral character. | .37/<.001 | .44/<.001 | - |  |  |
| 10. It is unfair if I must change my lifestyle to reduce my emissions if others don't do the same. | -.15/<.001 | -.33/<.001 | -.11/.008 | - |  |
| 11. Individuals are not to blame for climate change. | -.13/<.001 | -.21/<.001 | -.21/<.001 | .17/<.001 | - |
| 12. Tackling climate change is mainly the responsibility of governments and industries. | -.02/.672 | -.09/.020 | .06/.120 | .16/<.001 | .19/<.001 |
| *Note*. The items are numbered to continue from Table S4, to facilitate clearer discussion about the full set of items | | | | | |

| **Table S6**.  *Correlations/p-values between attitudinal and demographic variables and climate justice demands at international level.* | | | | | | |
| --- | --- | --- | --- | --- | --- | --- |
|  | 1. The countries that emit the most should help poor countries deal with the negative impacts of climate change. | 2. Rich countries should help poor countries deal with the negative impacts of climate change. | 3. Rich countries should help poor countries grow economically in ways that do not damage the environment. | 4. Sweden should offer asylum to people who need to leave their homes due to climate change. | 5. Sweden should not have to reduce emissions if this would hinder our economic development. | 6. Poor countries should not have to reduce their emissions if this would hinder their economic development |
| Climate worry, Personal | .17/<.001 | .18/<.001 | .19/<.001 | .22/<.001 | -.28/<.001 | -.11/.005 |
| Climate worry, Distant others | .40/<.001 | .44/<.001 | .42/<.001 | .35/<.001 | -.44/<.001 | -.04/.296 |
| Climate change denial | -.46/<.001 | -.52/<.001 | -.53/<.001 | -.40/<.001 | .55/<.001 | .10/.009 |
| Climate action | .29/<.001 | .40/<.001 | .35/<.001 | .50/<.001 | -.44/<.001 | -.02/.688 |
| Political trust | .14/<.001 | .14/<.001 | .17/<.001 | .15/<.001 | -.10/<.001 | .13/<.001 |
| Social Dominance Orientation (SDO) | -.37/<.001 | -.46/<.001 | -.41/<.001 | -.42/<.001 | .37/<.001 | .04/.334 |
| Right-Wing Authoritarianism (RWA) | -.22/<.001 | -.26/<.001 | -.27/<.001 | -.43/<.001 | .36/<.001 | -.07/.086 |
| Age | .13/<.001 | .18/<.001 | .20/<.001 | -.02/.562 | -.13/<.001 | -.12/.003 |
| Gender (male)^Ϯ^ | .01/.845 | -.02/.570 | -.07/.081 | -.14/<.001 | .14/<.001 | .01/.908 |
| Education level ^Ϯ^ | .07/.082 | .10/.009 | .12/.001 | .14/<.001 | -.14/<.001 | .03/.472 |
| *Note*. ^Ϯ^Spearmans Rho, other correlations: Pearson | | | | | | |

| **Table S7**.  *Correlations/p-values between attitudinal and demographic variables and climate justice demands at individual level.* | | | | | | |
| --- | --- | --- | --- | --- | --- | --- |
|  | 7. If other people reduce their emissions, I should do the same. | 8. I feel morally responsible for limiting my personal contribution to climate change. | 9. Individuals’ climate behaviors reveal their moral character. | 10. It is unfair if I must change my lifestyle to reduce my emissions if others don't do the same. | 11. Individuals are not to blame for climate change. | 12. Tackling climate change is mainly the responsibility of governments and industries. |
| Climate worry, Personal | .23/<.001 | .31/<.001 | .26/<.001 | -.04/.300 | -.21/<.001 | .06/.142 |
| Climate worry, Distant others | .40/<.001 | .49/<.001 | .32/<.001 | -.23/<.001 | -.20/<.001 | .04/.364 |
| Climate change denial | -.46/<.001 | -.52/<.001 | -.31/<.001 | .27/<.001 | .20/<.001 | -.06/.130 |
| Climate action | .41/<.001 | .54/<.001 | .39/<.001 | -.31/<.001 | -.17/<.001 | -.05/.198 |
| Political trust | .19/<.001 | .17/<.001 | .03/.451 | -.17/<.001 | -.05/.188 | .01/.784 |
| Social Dominance Orientation (SDO) | -.31/<.001 | -.37/<.001 | -.16/<.001 | .24/<.001 | .09/.025 | -.01/.720 |
| Right-Wing Authoritarianism (RWA) | -.20/<.001 | -.29/<.001 | -.03/.484 | .28/<.001 | .03/.397 | .01/.880 |
| Age | .08/.036 | .01/.744 | .09/.030 | -.14/<.001 | -.02/.581 | -.12/.002 |
| Gender (male)^Ϯ^ | -.14/<.001 | -.24/<.001 | -.11/.006 | .17/<.001 | .10/.011 | .03/.485 |
| Education level^Ϯ^ | .13/<.001 | .17/<.001 | .02/595 | -.12/.002 | -.02/.580 | -.01/828 |
| *Note*. ^Ϯ^Spearmans Rho, other correlations: Pearson | | | | | | |

| **Table S8**.  *Regression analyses predicting climate justice demands at international and Individual levels, reporting p-values and b-values (Including 95% CI).* | | | | |
| --- | --- | --- | --- | --- |
|  | Demands on behalf of poor countries | | Demands for individual climate action | |
|  | *b*(95% CI) | *p* | *b*(95% CI) | *p* |
| *Model 1* |  |  |  |  |
| Age | .009 (.006, .012) | <.001 | .004 (.001, .008) | .012 |
| Gender (male) | -.116 (-.241, .010) | .071 | -.374 (-.501, -.247) | <.001 |
| Education level | .074 (.029, .119) | <.001 | .067 (.022, .113) | .004 |
| *Model 2* |  |  |  |  |
| Political trust | .122 (.063, .181) | <.001 | .110 (.044, .175) | .001 |
| SDO | -.487 (-.566, -.407) | <.001 | -.359 (-.448, -.271) | <.001 |
| RWA | -.091 (-.170, -.012) | .024 | -.096 (-.184, -.008) | .032 |
| *Model 3* |  |  |  |  |
| Climate worry, Personal | -.105 (-.162, -.047) | <.001 | .030 (-.028, .088) | .311 |
| Climate worry, Distant | .213 (.143, .282) | <.001 | .148 (.078, .217) | <.001 |
| Climate change denial | -.431 (-.505, -.357) | <.001 | -.292 (-.367, -.217) | <.001 |
| Climate action | .166 (.083, .249) | <.001 | .406 (.323, .489) | <.001 |
| *Model 4* |  |  |  |  |
| Age | .006 (.003, .009) | <.001 | .002 (.000, .005) | .083 |
| Gender (male) | .080 (-.018, .179) | .109 | -.148 (-.252, -.043) | .006 |
| Education level | .019 (-.016, .054) | .288 | .015 (-.022, .051) | .437 |
| Political trust | .093 (.042, .144) | <.001 | .071 (.017, .125) | .010 |
| SDO | -.228 (-.306, -.151) | <.001 | -.023 (-.106, .059) | .578 |
| RWA | .034 (-.039, .106) | .365 | .042 (-.035, .119) | .285 |
| Climate worry, Personal | -.073 (-.130, -.017) | .011 | .017 (-.044, .077) | .588 |
| Climate worry, Distant | .166 (.099, .234) | <.001 | .143 (.071, .214) | <.001 |
| Climate change denial | -.356 (-.435, -.277) | <.001 | -.297 (-.381, -.213) | <.001 |
| Climate action | .127 (.045, .208) | .002 | .379 (.293, .465) | <.001 |
| *Note*. SDO=Social Dominance Orientation, RWA=Right-Wing Authoritarianism | | | | |
